# Systematic Analysis of Survival-Associated Alternative Splicing Signatures in Thyroid Carcinoma

**DOI:** 10.3389/fonc.2021.561457

**Published:** 2021-06-23

**Authors:** Baoai Han, Minlan Yang, Xiuping Yang, Mengzhi Liu, Qiang Xie, Guorun Fan, Davood K. Hosseini, Jintao Yu, Peng Song, Xiong Chen, Haiying Sun

**Affiliations:** ^1^ Department of Otorhinolaryngology, Union Hospital, Tongji Medical College, Huazhong University of Science and Technology, Wuhan, China; ^2^ Department of Otorhinolaryngology, Head and Neck Surgery, Zhongnan Hospital of Wuhan University, Wuhan, China; ^3^ Department of Internal Medicine, Hackensack University Medical Center, Hackensack, NJ, United States

**Keywords:** alternative splicing, thyroid carcinoma, prognosis, splicing factors, survival

## Abstract

Alternative splicing (AS) is a key mechanism involved in regulating gene expression and is closely related to tumorigenesis. The incidence of thyroid cancer (THCA) has increased during the past decade, and the role of AS in THCA is still unclear. Here, we used TCGA and to generate AS maps in patients with THCA. Univariate analysis revealed 825 AS events related to the survival of THCA. Five prognostic models of AA, AD, AT, ES, and ME events were obtained through lasso and multivariate analyses, and the final prediction model was established by integrating all the AS events in the five prediction models. Kaplan–Meier survival analysis revealed that the overall survival rate of patients in the high-risk group was significantly shorter than that of patients in the low-risk group. The ROC results revealed that the prognostic capabilities of each model at 3, 5, and 8 years were all greater than 0.7, and the final prognostic capabilities of the models were all greater than 0.9. By reviewing other databases and utilizing qPCR, we verified the established THCA gene model. In addition, gene set enrichment analysis showed that abnormal AS events might play key roles in tumor development and progression of THCA by participating in changes in molecular structure, homeostasis of the cell environment and in cell energy. Finally, a splicing correlation network was established to reveal the potential regulatory patterns between the predicted splicing factors and AS event candidates. In summary, AS should be considered an important prognostic indicator of THCA. Our results will help to elucidate the underlying mechanism of AS in the process of THCA tumorigenesis and broaden the prognostic and clinical application of molecular targeted therapy for THCA.

## Introduction

Alternative splicing (AS) is a posttranscriptional process. During the process of transforming precursor mRNA (pre-RNA) into mature mRNA, one gene can produce various mature mRNAs through multiple splicing and can be ultimately translated into different proteins with different functions. There are seven types of AS: alternate acceptor site (AA), alternate donor site (AD), alternate promoter (AP), alternate terminator (AT), exon jump (ES), exon mutual exclusion (ME), and intron retention (RI) (1). AS modifies more than 90% of human genes and is one of the most important mechanisms to enhance transcriptional diversity by changing the composition of exons in mRNA. Therefore, AS events are closely related to protein function (2).

Dysregulation of AS has been described in many disorders, especially tumorigenesis (3, 4). While isomers of “oncogenes” regulate the apoptosis of tumor cells, “tumor suppressor genes” regulate the migration and invasion ability of tumor cells. For instance, the apoptosis-related gene BCL-X encodes two protein isomers, BCL-XL and BCL-XS, through two different spliceosomes. These two isomers exhibit opposite effects on the regulation of apoptosis. BCL-XL inhibits apoptosis, while BCL-XS promotes apoptosis; BCL-XL is highly expressed in myeloid leukemia and indicates a poor prognosis, while BCL-XS is expressed at low levels in renal cancer, liver cancer, breast cancer, prostate cancer, and other tumor cell lines (5, 6). The proportions of Bcl-XL and Bcl-XS are regulated by splicing factors, such as SAM68, which can promote the expression of BCL-XS and induce apoptosis (7). A previous study showed that KAI1 is a tumor suppressor gene that can inhibit tumor metastasis. However, more recent studies have demonstrated that the isomer kai1-sp, which lacks exon 7, is involved in the metastasis of gastric cancer (8). In ovarian cancer, kai1-sp has been shown to be more highly expressed in metastatic cancer. Other studies have shown that this isomer has a carcinogenic effect and is correlated with the invasive ability of tumor cells (9). Several key splicing factors (SFs) regulate complex AS events. Changes in SF expression may lead to overall changes in some cancer-specific AS events, thus affecting the occurrence and development of cancer. As mentioned above, these results indicate that the potential splicing factor-alternative splicing (SF-AS) regulatory network may provide a new perspective for exploring biomarkers and the mechanisms of tumorigenesis.

Thyroid cancer is the seventh most common malignant tumor in women in the U.S (10) and has a sharply increasing incidence rate. Although thyroid cancer is inert in most cases, it is considered to be an important cause of morbidity and mortality due to the involvement of lymph nodes and distant metastases. Therefore, it is necessary to develop innovative diagnostic methods for the early detection and intervention of thyroid cancer. Abnormal selective splicing has been shown to be one of the important predisposing factors for thyroid cancer. However, few studies have performed a genome-wide analysis of the AS landscape of THCA patients. To clarify the contribution of AS events to the occurrence and development of THCA, we conducted a comprehensive analysis of seven AS events based on thyroid cancer. We aimed to determine the role of AS variants in the survival of patients with THCA and searched for key mechanisms that can accurately predict the prognosis of THCA patients.

## Materials and Methods

### Data Collation Process

The expression profiles and matching clinical information of the samples were downloaded from the TCGA database (**Supplementary Table 1**). First, we analyzed the batch effect in the data by TCGA Batch Effects Viewer (https://bioinformatics.mdanderson.org/public-software/) and no significant batch effect was found. Then, the clinical data were manually curated and cases with incomplete survival data were omitted from the downstream analysis. We finally obtained 58 non-tumor samples and 495 THCA samples in the analysis of AS events. In addition, the alternative splicing data was downloaded from the splicing sequence database (https://bioinformation.mdanderson.org/tcgaspliceq/index.jsp). The percent spliced index (PSI), an intuitive ratio to quantifying splicing events from 0 to 1, was calculated for seven types of AS patterns: alternate acceptor site (AA), alternate donor site (AD), alternate promoter (AP), alternate terminator (AT), exon skip (ES), mutually exclusive exons (ME), retained intron (RI). We removed the events that contained the vacancy values to make the results more reliable. The splicing factor (SF) gene list was collected from the SpliceAid 2 (http://193.206.120.249/splicing_tissue.html) and displayed in **Supplementary Table 2**.

### Identification of Survival-Related Events

Univariate Cox regression analysis was used to determine the relationship between AS events and overall survival (OS), and the threshold was set as P <0.05 (**Supplementary Table 3**). According to the seven AS event types, the interaction gene sets among the seven survival-related AS events were visualized quantitatively by Upset plot and volcano map. The parent genes of these survival related AS events were analyzed for Gene Ontology (GO) and Kyoto Encyclopedia of Genes and Genomes (KEGG) function enrichment. In addition, the bubble chart was used to summarize the top 20 AS events, top 20 AA events, top 20 AT events, top 20 AD events, top 20 AP events, top 20 ES events, top six ME events, and top 20 RI events.

### Construction of Prognostic Models of THCA Patients Based on AS Events

First of all, seven AS events related to prognosis were further analyzed by lasso regression analysis to screen out highly related AS events, so as to avoid overfitting of subsequent models. Then, multivariate Cox regression analysis was used to calculate the prognostic risk score for each AS event. The calculation formula is as follows: *β*1 * Exp1 + *β*2 * exp2 + *β*i * EXPi, in which *β* represents coefficient value and exp represents gene expression level. The risk score is an indicator to measure the prognostic risk of each THCA patient. All patients were divided into high-risk group and low-risk group according to the median risk score. Kaplan–Meier survival analysis was used to verify the statistical differences between low-risk and high-risk subgroups. The time-dependent receiver operating characteristic (ROC) curve evaluates the prediction ability of each AS signal. Area under the curve (AUC) value greater than or equal to 0.9 is a strong effective prediction value, greater than or equal to 0.70 is effective prediction value, greater than or equal to 0.6 is acceptable prediction value (11). In addition, the risk curve was used to evaluate the prognosis of risk score. The survival time of the expression thermogram, risk score distribution and related characteristics were visualized. The relationship between clinicopathological features such as age, sex, T stage, N stage, clinical stage, and survival of THCA patients was analyzed by using univariate and multivariate Cox regression forest plots to determine whether AS prognosis model is independent of other conventional clinical features.

### Comprehensive Analysis of AS Prognosis Model

Based on the five models of AA, AD, AT, ES, and ME events, we integrated all AS events in the five prediction models and established the final prediction model. The GEPIA database (http://GEPIA.cancerpku.cn/) was used to perform Kaplan–Meier analysis on the parental genes of AS events in the final prognosis model, and the log-rank test was used to determine the statistical significance. Then, in order to explore the characteristics and ways of enrichment in the predicted high- and low-risk population, we conducted gene set enrichment analysis (GSEA) for THCA patients based on the final prediction model. Using GSEA, this study examined whether the characteristics of activation/inhibition genes were abundant in high-risk and low-risk patients. The enrichment degree of the predefined signature and KEGG paths was calculated using standardized enrichment score (NES) and standardized P value. Terms with | NES |>1 and P <0.05 are considered significantly enriched.

### Verification of Screened AS Genes by qRT-PCR

Human thyroid follicular epithelial cells Nthy-ori 3-1 and THCA-derived cell lines (8505C and TPC1) were obtained from the Shanghai Zhong Qiao Xin Zhou Biotechnology. Nthy-ori 3-1 was cultivated in RPMI-1640 medium, while TPC1 and 8505C cells were maintained in Dulbecco’s modified Eagle’s medium (DMEM), all media containing 10% fetal bovine serum (FBS) (Biological Industries, Kibbutz Beit Haemek, Israel) and 1% penicillin–streptomycin (Keygen Biotech, Nanjing, China), and incubated at 37°C in a humidified atmosphere containing 5% CO_2_. For quantitative analyses of screened AS genes, total RNA was extracted using TrizoL (Life Technologies, Carlsbad), and cDNA was prepared using High-Capacity cDNA Reverse Transcription Kit (Thermo Fisher, MA), and qPCR was performed with NovoStart^®^ SYBR qPCR SuperMix Plus (Novoprotein, Shanghai, China). The qPCR primer sequences of POLM gene were 5′-ACTTTGGAGAACACTCCTCTAGG-3′ and 5′-GCAGTCTTCACACCGACCC-3′, primer sequences of ZBTB45 were 5′-GACTGTGCGCATTCGTGAAG-3′ and 5′-CGAACCGCTGTACAGGAACT-3′, primer sequences of FAM185A were 5′-CACTGCAGGGTCAAAAATTGC-3′ and 5′-ACTTGGCTTTCAGCAAACCATC-3′, primer sequences of C12orf57 were 5′-CAACGACATGGGTGTCCTTAAGTT-3’ and 5’-GTCATGGGCGGCAGAAACAG-3’, which were designed by Primer Premier 5. The expression of cDNA samples was verified using GAPDH as a housekeeping gene.

### Construction of Splicing Factor Control Network

Seventy-one splicing factor (SF) gene lists were collected from the splicing assistant 2 database, and the expression profile of SF gene was downloaded from the TCGA database. Spearman correlation method was used to calculate the correlation coefficient between the PSI value and SF expression value of AS events related to survival. Finally, we also build the interaction network of AS and SF. The splicing-related network was built and drawn by the software of Cytoscape (version 3.4.0). In this section, R software (version = 3.5.3) was used for all statistical analyses. P value less than 0.05 is considered statistically significant.

## Results

### Overview of AS Profiling in THCA Cohort

**Figure 1:**
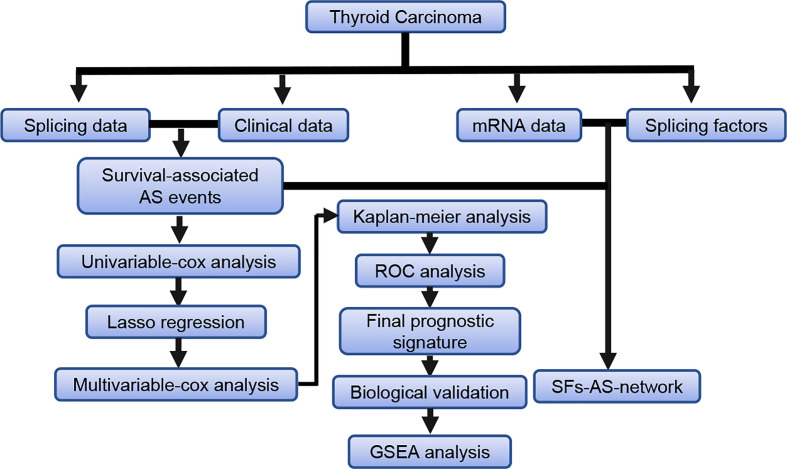
The flowchart for profiling the AS events in thyroid carcinoma.

**Figure 2:**
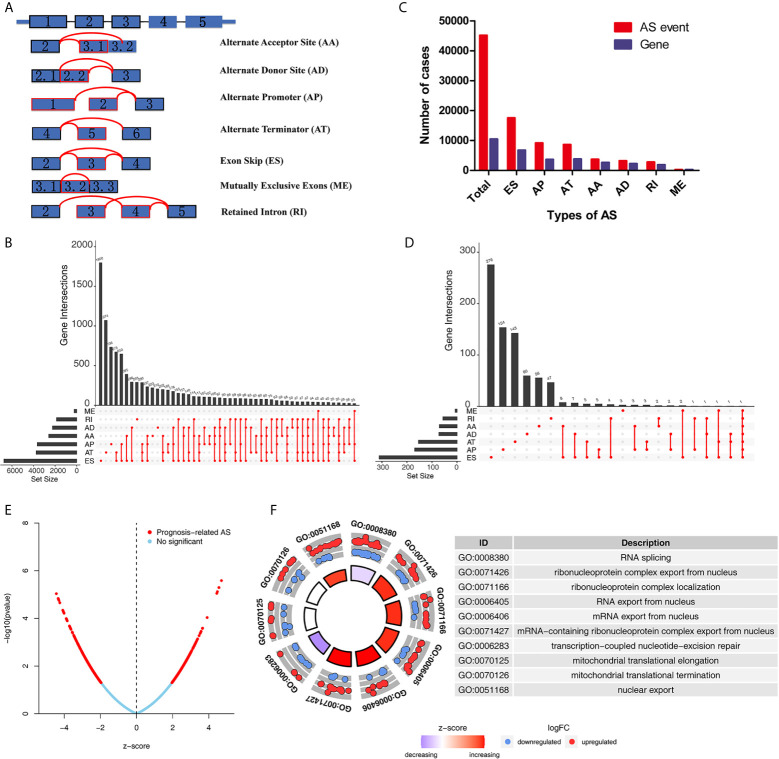
Overview of AS events and Gene Ontology (GO) functional annotation analysis in TCGA THCA cohort. **(A)** Seven types of AS events were illustrated including exon skip (ES), retained intron (RI), alternate promoter (AP), alternate terminator (AT), alternate donor site (AD), alternate acceptor site (AA), and mutually exclusive exons (ME). **(B)** Numbers of AS events and AS-associated genes in THCA patients. **(C)** Upset plot of interactions among seven types of all AS events in THCA. **(D)** Upset plot of interactions among seven types of survival-associated AS events in THCA. **(E)** Volcano plots of prognostic AS events. **(F)** Results of GO functional annotation analysis. The outer circle shows a scatter plot for each term of the logFC of the assigned genes. Red circles display up-regulation pathways, and blue circles showing the down-regulation pathway. AS, alternative splicing; THCA, Thyroid Carcinoma; TCGA, The Cancer Genome Atlas.

The detailed workflow information of the study was shown in **Figure 1**. First, we combined the AS data and clinical information (**Table 1**) of patients with THCA to obtain survival-related AS events, and then we established a variety of prognostic models of variable AS events through univariate analysis, lasso regression, and multivariate analysis and further used the KM method and time-dependent ROC analysis to verify the predictive power of these models. Finally, we analyzed the regulation of the splicing factor on variable AS events and verified the screened genes *via* qPCR. The seven splicing types of the AS event include alternate acceptor site (AA), and alternate donor site (AD), alternate promoter (AP), alternate terminator (AT), exon skip (ES), mutually exclusive exons (ME), and retained intron (RI) (**Figure 2A**). A total of 45,150 AS events from 10,447 genes were detected by AS pattern analysis in 495 THCA patients. Among them, there are 3,683 AA in 2,592 genes, 3,190 AD in 2,240 genes, 9,127 AP in 3,653 genes, 8,595 AT in 3,753 genes, 17,536 ES in 6,748 genes, 232 ME in 224 genes, and 2,787 RI in 1,865 genes (**Figure 2B**). In addition, we utilized upset plot to quantitatively analyze the intersections and interaction sets among seven types of AS events (**Figure 2C**). These data suggest that ES event is the main type of AS in THCA patients, and about 39% are ES splicing event.

**Table 1:** Clinicopathological characteristic of 507 patients with thyroid carcinoma from TCGA database.

Clinical parameters	Variable	Total (507)	Percentages (%)
**Age**	<=65	436	86.0%
	>65	71	14.0%
**Gender**	Female	371	73.2%
	Male	136	26.8%
**Pathological stage**	Stage I	285	56.2%
	Stage II	52	10.3%
	Stage III	113	22.3%
	Stage IV	55	10.8%
	Unknow	2	0.4%
**T stage**	T1	144	28.4%
	T2	167	32.9%
	T3	171	33.7%
	T4	23	4.5%
	TX	2	0.4%
**M stage**	M0	283	55.8%
	M1	9	1.8%
	MX	214	42.2%
	Unknow	1	0.2%
**N stage**	N0	231	45.6%
	N1	226	44.6%
	NX	50	9.9%
**Survival status**	Dead	16	3.2%
	Alive	491	96.8%

T, tumor; M, metastasis; N, node (regional lymph node).

### Identify Survival-Related AS Events in THCA

**Figure 3:**
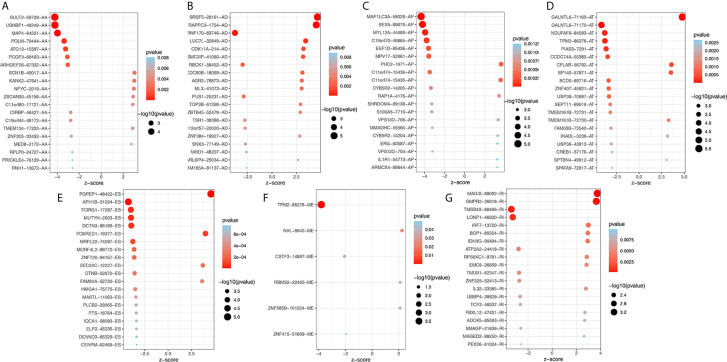
Bubble plots of top 20 significant prognostic AS events in AA **(A)**, AD **(B)**, AP **(C)**, AT **(D)**, ES **(E)**, ME **(F)** and RI **(G)** type, respectively. AA, alternate acceptor site; AD, alternate donor site; AP, alternate promoter; AT, alternate terminator; ES, exon skip; ME, mutually exclusive exons; RI, retained intron.

To explore the potential association between AS events and overall survival (OS) in THCA patients, we performed a univariate Cox analysis to determine survival-related AS events in THCA cohort. A total of 933 survival-related AS events from 784 parental genes were identified in THCA (**Supplementary Table 3**) which suggested that the given gene may contain two or more AS events that are significantly associated with OS. The upset plot was generated to vividly show the intersection set of genes and splicing types (**Figure 2D**) while the distribution of AS events was found to be significantly related to OS (**Figure 2E**). Moreover, functional enrichment analysis on the parental genes of AS events related to survival prognosis revealed that the most abundant GO items in biological process include RNA splicing, ribonucleoprotein complex export from nucleus, and ribonucleoprotein complex localization (**Figure 2F**). Cell components include mitochondrial matrix, mitochondrial inner membrane, and organellar ribosome. In terms of molecular functions, genes were mostly enriched in DNA polymerase binding and single-stranded DNA binding. **Figure 3** selects and shows the top 20 (if available) significant survival rates associated with each type of event. 

### Construction and Evaluation of AS Prognostic Markers

**Figure 4:**
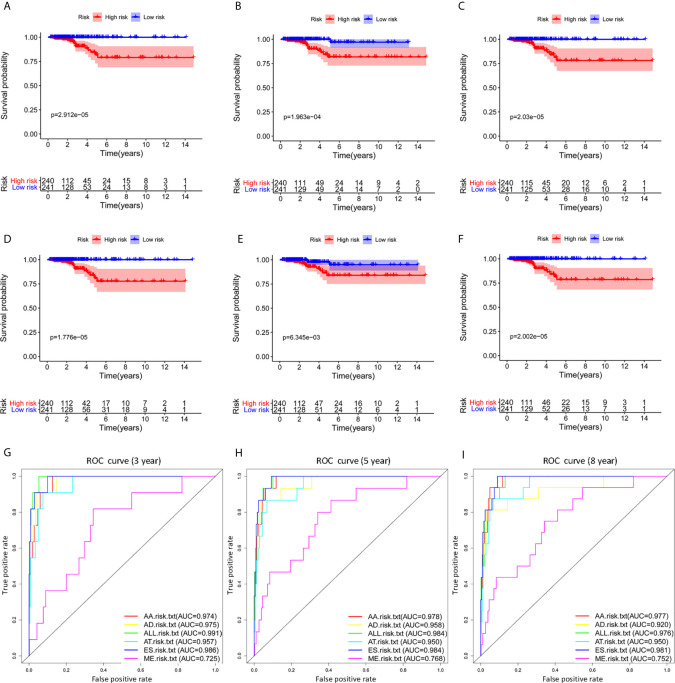
The Kaplan–Meier curves analysis of overall survival between the low-risk patients and high-risk patients. AA cohort **(A)**, AD cohort **(B)**, AT cohort **(C)**, ES cohort **(D)**, ME cohort **(E)**, the final prediction cohort **(F)**, **(G–I)** The 3, 5 and 8 year ROC curves of prognostic models based on individual type or all types of AS events in THCA. THCA, thyroid carcinoma; ROC, receiver operating characteristic.

**Figure 5:**
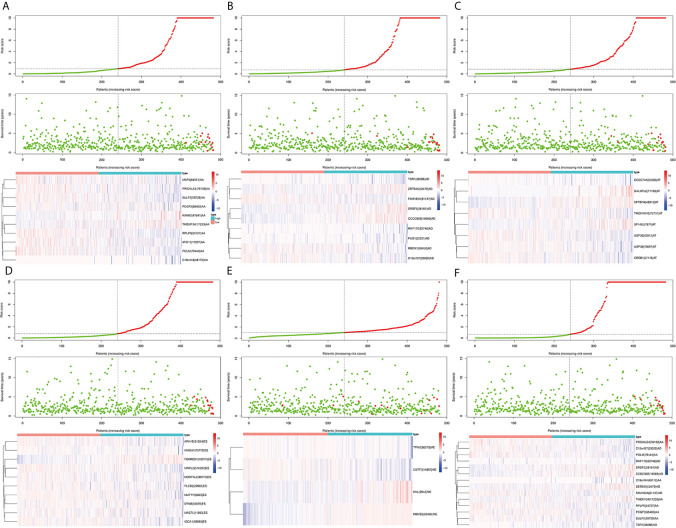
The distribution of risk score (upper panel), the distribution of survival time (middle panel), the expression Heatmap (bottom panel) of six significant prognostic signatures. AA cohort **(A)**, AD cohort **(B)**, AT cohort **(C)**, ES cohort **(D)**, ME cohort **(E)**, the final prediction cohort **(F)**.

Survival related AS events were screened out by univariate Cox regression analysis, and the most significant AS events were further selected by lasso regression analysis (**Supplementary Figure 1**). Finally, predictive models based on each AS event type were constructed by multivariate Cox regression analysis. Then, the prediction models of five AS events of AA, AD, AT, ES, and ME were obtained. Among the AP and RI AS events, no prediction model was obtained since there were not enough genes after lasso regression analysis. All AS events in the five prediction models are integrated to establish the final prediction model (**Table 2**). Kaplan–Meier analysis showed that these prognostic models effectively stratified patients with different prognoses, and the OS of patients in the high-risk group was significantly shorter than that in the low-risk group (**Figures 4A–F**). In order to compare the prediction capabilities of these prediction models in 3, 5, and 8 years, we further applied ROC analysis. The data demonstrated that all models have satisfactory predictive performance, with AUC values ranging from 0.725 to 0.991 (**Figures 4G–I**). The prediction model of combining five AS events has better prediction performance than the prediction model of individual AS events. The AUC values are 0.991, 0.984, and 0.976 in the 3, 5, and 8 years, respectively. (**Figure 5** shows the details of the event, survival status, survival time, candidate gene splicing patterns sorted by risk score distribution).

**Table 2:** Multivariate Cox analysis of prognostic alternative splicing predicting overall survival.

Gene symbol	Gene ID	AS pattern	Coef	HR	HR.95L	HR.95H	P value	Final model
SULF2	59729	AA	−1.0068	0.3654	0.1935	0.6901	0.0019	*
MAP4	64551	AA	−0.6743	0.5095	0.3267	0.7945	0.0029	
POLM	79444	AA	−1.0753	0.3412	0.1824	0.6383	0.0008	*
ATG13	15597	AA	−0.8668	0.4203	0.2535	0.6969	0.0008	
PCGF3	68403	AA	−0.5332	0.5867	0.3497	0.9844	0.0434	*
KANK2	47641	AA	0.4517	1.5710	0.9957	2.4787	0.0522	
C19orf44	48172	AA	−0.7102	0.4915	0.3681	0.6564	0.0000	*
TMEM134	17233	AA	0.7640	2.1469	1.2167	3.7883	0.0084	*
RPLP0	24727	AA	−1.1704	0.3102	0.1431	0.6728	0.0030	*
PRICKLE4	76139	AA	−0.4467	0.6397	0.3766	1.0867	0.0985	*
SRSF5	28161	AD	1.1989	3.3166	2.0069	5.4809	0.0000	*
RNF170	83746	AD	−0.4729	0.6232	0.4809	0.8075	0.0003	*
RBCK1	58452	AD	−0.6923	0.5004	0.2971	0.8430	0.0093	
CCDC90B	18069	AD	1.0031	2.7268	1.5120	4.9175	0.0009	*
PUS1	25231	AD	−0.3403	0.7116	0.4806	1.0535	0.0892	
ZBTB45	52479	AD	0.5486	1.7308	0.9734	3.0778	0.0618	*
TSR1	38388	AD	−0.9975	0.3688	0.2157	0.6305	0.0003	*
C12orf57	20020	AD	−1.0084	0.3648	0.1888	0.7049	0.0027	*
FAM185A	81137	AD	−0.7857	0.4558	0.2483	0.8366	0.0112	*
GALNTL6	71169	AT	0.3139	1.3688	0.9571	1.9575	0.0855	
CCDC74A	55385	AT	−0.4095	0.6640	0.5437	0.8109	0.0001	
SP140	57871	AT	0.9347	2.5464	1.5637	4.1466	0.0002	
USP38	70697	AT	−0.7792	0.4588	0.3097	0.6795	0.0001	
TMEM161B	72731	AT	−1.0285	0.3575	0.1938	0.6596	0.0010	
USP36	43913	AT	−0.7703	0.4629	0.2599	0.8245	0.0089	
CREB1	57176	AT	−0.9615	0.3823	0.2213	0.6606	0.0006	
SPTBN4	49912	AT	1.0888	2.9708	1.4375	6.1396	0.0033	
APH1B	31024	ES	−0.6085	0.5442	0.2849	1.0392	0.0653	
MUTYH	2603	ES	−0.8228	0.4392	0.2829	0.6818	0.0002	
FOXRED1	19377	ES	1.9049	6.7184	2.6887	16.7881	0.0000	
MRPL22	74297	ES	−1.2591	0.2839	0.1397	0.5771	0.0005	
MORF4L2	89772	ES	−0.6965	0.4983	0.3000	0.8278	0.0072	
DTNB	52870	ES	−0.3701	0.6907	0.4786	0.9967	0.0479	
HMGA1	75775	ES	−0.5247	0.5917	0.4588	0.7631	0.0001	
MASTL	11063	ES	−0.6376	0.5285	0.3623	0.7710	0.0009	
PLCB2	29965	ES	−0.4919	0.6115	0.3869	0.9664	0.0352	
IQCA1	58093	ES	−0.8100	0.4449	0.2466	0.8024	0.0071	
TPM2	86278	ME	−0.2228	0.8002	0.6765	0.9467	0.0093	
NVL	9942	ME	0.3976	1.4883	1.0354	2.1391	0.0317	
CSTF3	14887	ME	−0.3736	0.6882	0.4378	1.0820	0.1056	
RBMS2	22465	ME	0.6483	1.9123	0.9078	4.0284	0.0881	

HR, hazard ratio; HR.95 L/H, 95% confidence interval of the hazard ratio. *Genes selected in the final prediction model.

### Comprehensive Analysis of Prognostic Models

**Figure 6:**
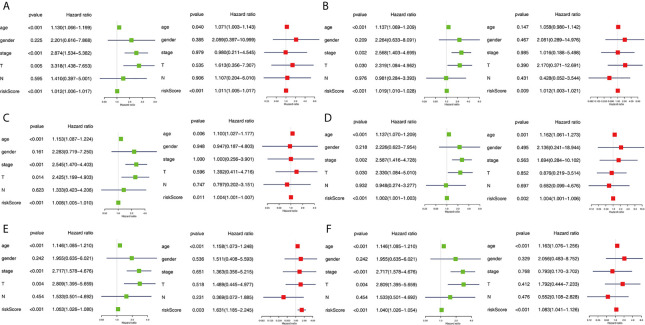
Independent prognostic analysis determining predictors of overall survival. AA cohort **(A)**, AD cohort **(B)**, AT cohort **(C)**, ES cohort **(D)**, ME cohort **(E)**, the final prediction cohort **(F)**. Forest plots showing the univariate (left) and multivariate cox regression analyses (right) of OS in THCA.

**Figure 7:**
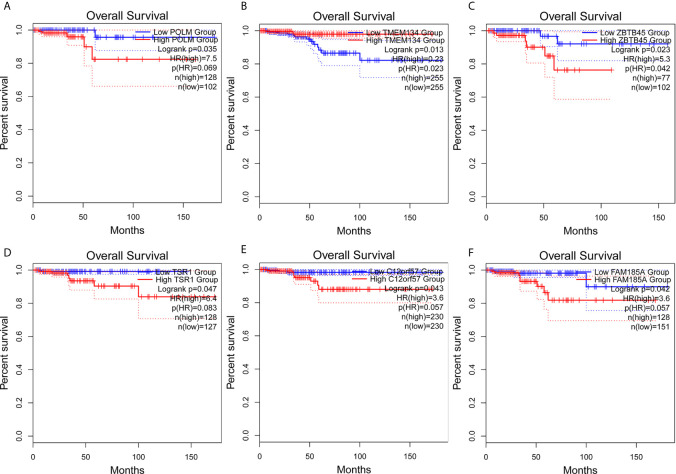
Survival analysis of AS parent genes in the final prediction model. Kaplan**–**Meier analyses of **(A)** POLM, **(B)** TMEM134, **(C)** ZBTB45, **(D)** TSR1, **(E)** C12orf57, **(F)** FAM185A. The statistical significance was determined by *log-rank* test.

**Figure 8:**
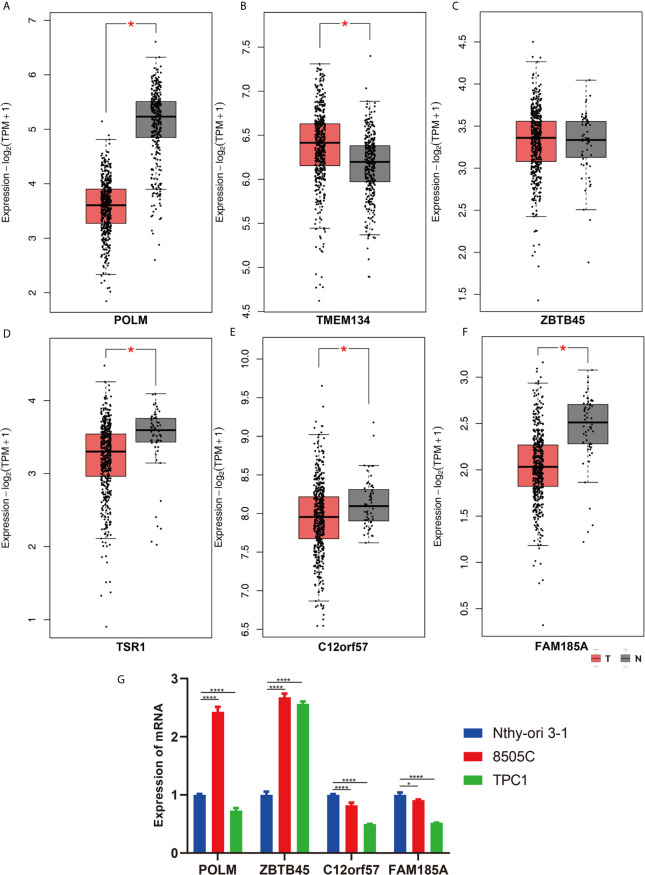
The validation of screened genes in the final prediction model. The expression difference of **(A)** POLM, **(B)** TMEM134, **(C)** ZBTB45, **(D)** TSR1, **(E)** C12orf57, **(F)** FAM185A between THCA and Control in GPEIA2 database. **(G)** The relative mRNA expression of screened genes was evaluated by qRT-PCR. *p < 0.05; ****p < 0.0001.

**Figure 9:**
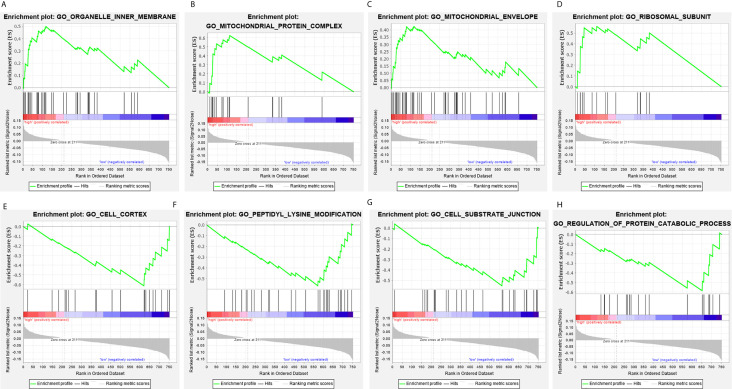
Gene set enrichment analysis of genes in high-risk and low-risk patients in THCA. **(A–D)** Gene set enrichment analysis (GSEA) showing the enrichment of hallmarks in high-risk patients with THCA. **(E–H)** GSEA showing the enrichment of hallmarks in low-risk patients with THCA.

The clinical pathological parameters such as gender, T stage, N stage, and clinical stage were included in the univariate/multivariate Cox regression analysis. Univariate Cox regression analysis showed that the advanced age, high T stage, high clinical stage and high risk score can predict the overall survival rate of THCA patients. However, multivariate Cox regression showed that only age and risk score were independent risk factors for THCA survival (**Figure 6**). In order to further explore whether the genes related to AS provide clinical significance, we analyzed the significance of the survival of the parent gene in the final prediction model. The results showed that most genes have survival significance in THCA (**Figure 7**). To verify our data, we first verified the expression difference of the selected genes between thyroid cancer and control group in GPEIA2 database (**Figures 8A–F**), then the relative mRNA expression of screened genes was evaluated by qRT-PCR. Finally, we found that four genes were consistent with our data. As shown in **Figure 8G**, compared with that in the normal thyroid epithelial cell line Nthy-ori 3-1, the mRNA expression of the POLM was higher in 8505C cells and lower in TPC1 cells (P < 0.01); the ZBTB45 mRNA expression was higher in both 8505C and TPC1 cells (P < 0.01), and the C12orf57 and FAM185A mRNA expressions were lower in both 8505C and TPC1 cells (P < 0.01). These results further verified the reliability of the model. We found that in the final prediction model, high-risk and low-risk patients have significant prognostic differences in OS; GSEA was used to explain the rich features and pathways between high-risk and low-risk patients. It revealed that the high-risk group was enriched in organelle intima membrane, mitochondrial protein complex, mitochondrial membrane, ribosome subunit, and other ways (**Figures 9A–D**). The low-risk group was enriched in cell cortex, peptide lysine modification, cell substrate junction, regulation of protein catabolic process, and other ways (**Figures 9E–H**). The results of GSEA analysis suggest that AS event-related signals may be related to the development and progression of THCA.

### Construction of Splicing Regulatory Network

**Figure 10:**
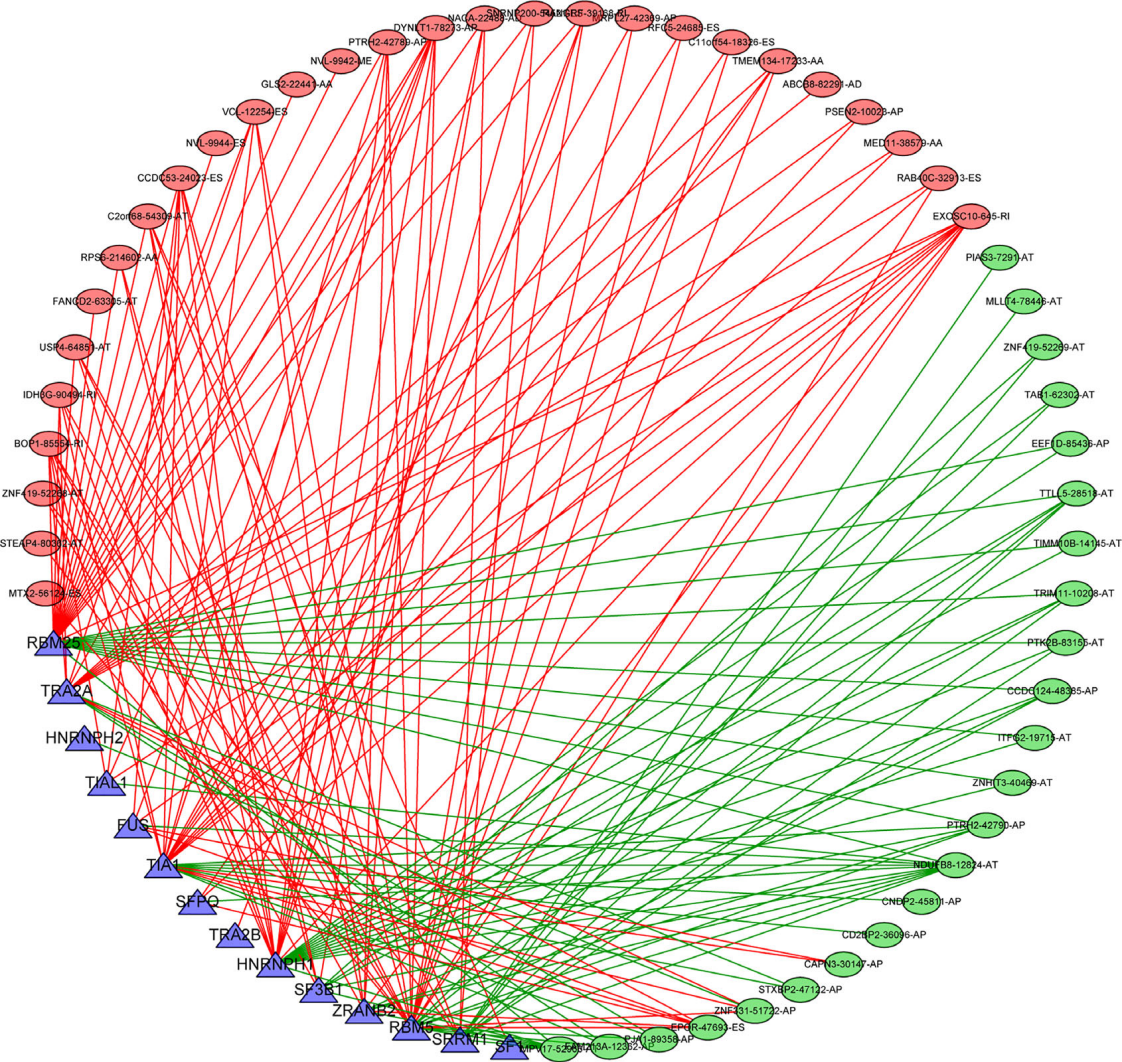
Splicing correlation network in THCA. Seven survival-related SFs (blue triangle) were positively (green lines)/negatively (red lines) correlated with PSI values of survival-related AS events with favorable prognosis (green oval) or survival-related AS events with poor prognosis (red oval). SFs, splicing factor; AS, alternative splicing; THCA, thyroid carcinoma.

Splicing factors are RNA binding proteins that affect both exon selection and splicing site selection by recognizing *cis* regulatory elements in pre mRNA. Therefore, we inferred that these survival-related AS events may be regulated by certain key SFs. To address this issue, we explored the potential association between survival-related AS events and SFs. By retrieving the expression of SFs in the TCGA-THCA data set, a regulatory network of SF expression and survival-related AS events was established. As shown in **Figure 10**, there were totally 51 AS events that were significantly related to the expression level of these 14 SFs (blue triangle), 23 of which had a good prognosis (green oval) and 28 had a poor prognosis (red oval) (**Supplementary Tables 4**, **5**). It is worth noting that different SFs can have a positive or negative regulatory effect on the same AS events simultaneously, while abnormal AS events are regulated by the synergistic or competitive effects of different prognosis SFs. These results further illustrate the potential mechanism for AS to have great potential in expanding transcript encoding capacity. On the other hand, it is interesting that most AS events with good prognosis (blue dots) are negatively correlated with the expression of SFs (green line), while most AS events with poor prognosis (red dots) are positively correlated with SFs (red line).

## Discussion

The prognosis of patients with THCA is relatively good, but traditional antitumor therapy cannot achieve satisfactory results because of radiation-resistant papillary carcinoma, undifferentiated carcinoma, and medullary thyroid carcinoma (12). Although an increasing number of studies have demonstrated the relationship between AS and THCA, applying AS to the clinical treatment of THCA requires further investigation. AS allows a gene to produce multiple mRNA transcripts, which provide diversity of protein structure and function. High-throughput sequencing technology can be used to identify various biomarkers at the gene level, such as new alternative splicing events, which are closely related to the survival of patients (13). In recent decades, a large number of studies have confirmed that AS disorders promote the occurrence and development of cancer by dysregulating cell proliferation, invasion, metastasis, apoptosis, drug dependence, and immune escape (14–16). For instance, tyrosine kinase inhibitors (TKIs) are used to treat leukemia with a BCR-ABL fusion gene, and abnormally spliced mRNA precursors after BCR-ABL transcription are often found that lack the target domain of TKIs and allow tumor cells to escape killing by drugs (17, 18). In melanomas with the BRAF (V600E) mutation, the effective rate of using the RAF inhibitor verofinib is more than 50%. However, after approximately six months of treatment, the vast majority of patients developed drug resistance. Melanoma cells produce isomers that lack exons 4**–**8 of BRAF (V600E), which allows them to avoid the effects of drugs (19, 20). Thus, abnormal AS may play a role in the development of THCA. Here, we utilized THCA data from TCGA and combined them with bioinformatics analysis to identify AS events related to the prognosis of THCA and to develop a prognostic model of THCA.

We systematically identified and analyzed survival-related AS events in THCA samples and found 825 survival-related AS events in 719 genes. Through GO analysis of the parental genes of these events, we found that the enrichment events were mainly in the pathways of “RNA splicing”, “mitochondrial matrix”, and “mitochondrial inner membrane”. RNA splicing-related items were significantly enriched, which might highlight the involvement of the transesterification reaction and spliceosome-related mechanism in the abnormal splicing mode of THCA. In addition, the abnormal accumulation of mitochondrial-related terms suggests that cancer-related mechanisms may involve interfering with processes related to energy production, transmission, and utilization. To explore the prognostic significance of AS events, we built a prediction model for each AS event in thyroid cancer and found five models based on AA, AD, AT, ES, and ME events. Finally, we integrated all the AS events from the five prediction models and established the final prediction model. Various other databases were searched and qPCR verification was performed, and most of the model genes identified were consistent with our previous results, which verified the reliability of the model. According to the risk score, patients were divided into low-risk and high-risk groups. Kaplan**–**Meier analysis showed a significant difference in overall survival between low-risk and high-risk patients. The ROC results showed that the predictive powers of 3, 5, and 8 years were greater than 0.9 except for ME, and the predictive power of the model composed of ME events was greater than 0.7, which is a moderate-intensity prediction, indicating that these AS events have important predictive significance for the survival outcome of THCA patients. GSEA was conducted to explore the specific pathways involved in the 14 candidate survival-related AS events in the final prediction model. Interestingly, signals such as organelle intima, mitochondrial protein complex, mitochondrial membrane, and ribosome subunit were significantly overexpressed in high-risk individuals, while the low-risk group was mainly enriched in cell cortex, peptide lysine modifications, cell substrate binding, protein metabolism process regulation, and so on. Abnormal mitochondrial function is closely related to the occurrence and progression of tumors (21–23), suggesting that abnormalities in the structure and function of mitochondria may be the cause of THCA. Mitochondria produce the energy required to perform processes such as cell division, growth, and cell death. Functionally impaired mitochondria trigger disorder of the intracellular environment, which affects the normal performance of mitochondria, thereby inducing the malignant transformation of normal cells. Hyperproliferating cancer cells undergo different physiological processes, such as mitochondrial oxidative phosphorylation dysfunctions and changes in the cell metabolism enzyme spectrum (24–26), which lead to abnormal mitochondrial function.

In addition, although metabolic changes in tumor cells may not be the main cause of tumor initiation, changes in the tumor microenvironment are conducive to the growth of tumor cells, which has very important clinical and basic research value (27, 28). The group of AS events we identified exerts their biological functions in the energy metabolism of thyroid cancer. However, to the best of our knowledge, there are few studies on AS events in THCA, and research on these six AS types may have important predictive value in the future. Given the high prevalence of splicing defects in tumors, the prognostic events identified in this study may be new targets for THCA treatment. Through comprehensive analysis of the prognostic models, the selected genes showed strong effects on survival, and the expression of four selected genes, POLM, ZBTB45, C12orf57, and FAM185A, was consistent with our prediction, which confirmed the accuracy of our final prediction model.

There is increasing evidence that AS events may be regulated by key SFs. SF expression or sequence mutations may be an important mechanism of tumor splicing deregulation. To clarify the potential mechanism of AS events in the survival of THCA patients, we established an interaction network between AS events and SFs. This network revealed that the splicing factor RBM25 was closely related to the network and might contribute to the prognosis of splicing events. It has been reported that RBM25 regulates the retention of most selective exons in the human genome and interacts with the early spliceosome component SF3B and various splicing regulators (29, 30). The RNA sequencing results showed that RBM25 was involved in the regulation of multiple splicing events, including exon skipping and intron selection of retention and splice sites. RBM25 inhibits cell proliferation by inducing apoptosis and regulating the cell cycle in multiple cancers (31, 32). However, the role of RBM25 in THCA has not been studied. Therefore, exploring the molecular mechanism of RBM25 in splicing regulation may lead to the development of new treatments for thyroid cancer. In addition, the SF**–**AS regulatory network showed that most AS events with poor prognosis were positively correlated with SF. It is conceivable that overexpression of SFs may promote the occurrence of AS events and lead to a poor prognosis. The relationship between selected SFs and AS events and their potential role in AS generation in the occurrence and development of THCA are largely unknown. Recently, immune therapy has shown strong antitumor activity for the treatment of solid tumors, such as melanoma, non-small cell lung cancer, kidney cancer, and prostate cancer. Using mass spectrometry analysis, a large number of antigen peptides on the surface of tumor cells can be identified, and then, tumor-specific alternative splicing neoantigens can be screened. AS leads to mutations in key genes during cancer progression (33). The occurrence of variable splicing events in different tumors is tumor-specific. It has been found that some tumor-specific alternative splicing target antigens, such as EGFR alternative splicing, only exist in some tumor cells (34). The neoantigens derived from alternative splice variants greatly expand the tumor-specific target antigen library and allow the screening of tumor tissue-specific and highly immunogenic neoantigens as potential targets for cellular immunotherapy, and incorporating mRNA AS-derived neoepitopes as potential targets for anticancer approaches may allow THCA patients to benefit from such treatments. The occurrence and development of thyroid carcinoma are complex. Exonic alterations at the DNA level (especially before splicing sites), even polymorphic alterations, may play critical roles as predisposing factors through the initiation and progression phases of thyroid carcinoma. The effect of AS on thyroid carcinoma at the single-cell level has also been studied. In addition, the family history of thyroid carcinoma and other neoplastic disorders in patients’ pedigrees is important for cancer diagnosis and prevention. Therefore, more *in vivo* and *in vitro* functional experiments are needed to further explore the effects of dysregulated AS events and SFs on cancer development. In conclusion, further study of AS events will facilitate the application of complementary strategies for determining prognosis and early detection as well as the development of more effective therapies in the future.

## Data Availability Statement

The original contributions presented in the study are included in the article/**Supplementary Material**. Further inquiries can be directed to the corresponding authors.

## Author Contributions

HS, XC, and PS designed the research. BH, XY, MY, QX, and JY analyzed the data. GF and DH analyzed statistical data. XC and GF performed functional analysis. BH, XY, and ML wrote the first draft of the paper. HS, XC, and PS modified the language in the revisions. BH and XY prepared all the figures. All authors contributed to the article and approved the submitted version.

## Funding

This research was supported by grants from the National Natural Science Foundation of China (81600801, 81570903, 81600807, 81902599).

## Conflict of Interest

The authors declare that the research was conducted in the absence of any commercial or financial relationships that could be construed as a potential conflict of interest.

## Supplementary Material

Supplementary Figure 1 Lasso regression analysis of survival-associated AS events. AA cohort **(A)**, AD cohort **(B)**, AP cohort **(C)**, AT cohort **(D)**, ES cohort **(E)**, ME cohort **(F)** and RI cohort **(G)**, the final prediction cohort **(H)**. Left, Dotted vertical lines were drawn at the optimal values by using the minimum criteria. AS, alternative splicing; LASSO, least absolute shrinkage and selection operator. Right, LASSO coefficient profiles of the candidate survival-related AS events. A coefficient profile plot was produced against the log* λ* sequence.

Supplementary Table 1 Clinicopathological parameters of all patients in THCA.

Supplementary Table 2 SF list.

Supplementary Table 3 Univariate analysis results.

Supplementary Table 4 corResult.txt default edge.

Supplementary Table 5 corResult.txt default node.
