# Supplementary figures and images for: Systematic Analysis of Survival-Associated Alternative Splicing Signatures in Thyroid Carcinoma
Source: Front Oncol. 2021 Jun 23;11:561457. doi: 10.3389/fonc.2021.561457 (PMC8261059; doi:10.3389/fonc.2021.561457)

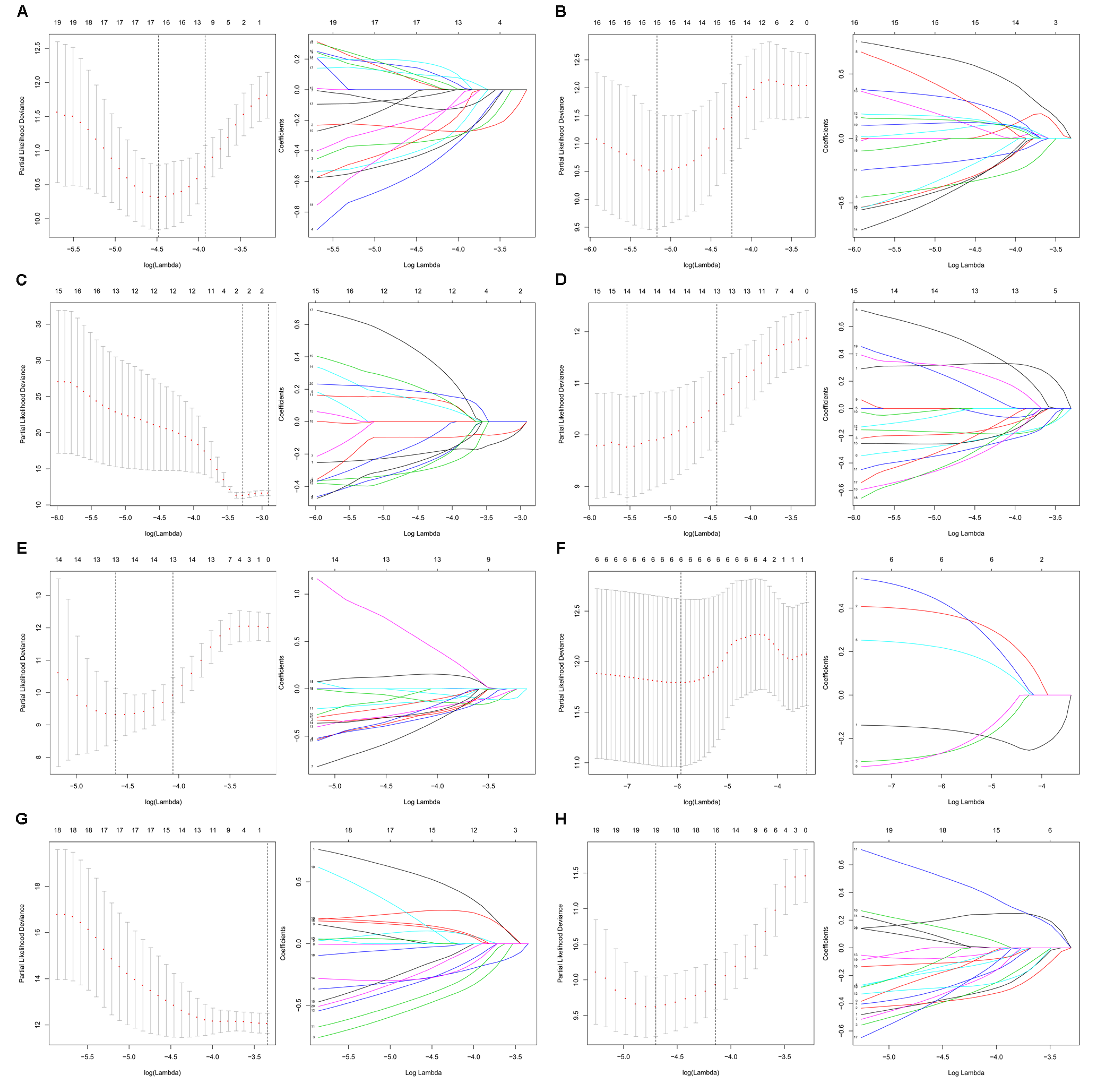

Supplement: Supplementary Figure 1 — Lasso regression analysis of survival-associated AS events. AA cohort (A), AD cohort (B), AP cohort (C), AT cohort (D), ES cohort (E), ME cohort (F) and RI cohort (G), the final prediction cohort (H). Left, Dotted vertical lines were drawn at the optimal values by using the minimum criteria. AS, alternative splicing; LASSO, least absolute shrinkage and selection operator. Right, LASSO coefficient profiles of the candidate survival-related AS events. A coefficient profile plot was produced against the log λ sequence. [file Image_1.tif]
